# The biochemical and molecular investigation of flower color and scent sheds lights on further genetic modification of ornamental traits in *Clivia miniata*

**DOI:** 10.1093/hr/uhac114

**Published:** 2022-05-17

**Authors:** Yueqing Li, Ruifang Gao, Jia Zhang, Yanan Wang, Peiru Kong, Keyu Lu, Adnan, Meng Liu, Feng Ao, Chunli Zhao, Li Wang, Xiang Gao

**Affiliations:** Key Laboratory of Molecular Epigenetics of MOE and Institute of Genetics & Cytology, Northeast Normal University, Changchun 130024, China; Key Laboratory of Molecular Epigenetics of MOE and Institute of Genetics & Cytology, Northeast Normal University, Changchun 130024, China; Key Laboratory of Molecular Epigenetics of MOE and Institute of Genetics & Cytology, Northeast Normal University, Changchun 130024, China; Key Laboratory of Molecular Epigenetics of MOE and Institute of Genetics & Cytology, Northeast Normal University, Changchun 130024, China; Key Laboratory of Molecular Epigenetics of MOE and Institute of Genetics & Cytology, Northeast Normal University, Changchun 130024, China; Key Laboratory of Molecular Epigenetics of MOE and Institute of Genetics & Cytology, Northeast Normal University, Changchun 130024, China; Key Laboratory of Molecular Epigenetics of MOE and Institute of Genetics & Cytology, Northeast Normal University, Changchun 130024, China; Key Laboratory of Molecular Epigenetics of MOE and Institute of Genetics & Cytology, Northeast Normal University, Changchun 130024, China; Key Laboratory of Molecular Epigenetics of MOE and Institute of Genetics & Cytology, Northeast Normal University, Changchun 130024, China; College of Horticulture, Jilin Agricultural University, Changchun 130118, China; Key Laboratory of Molecular Epigenetics of MOE and Institute of Genetics & Cytology, Northeast Normal University, Changchun 130024, China; Key Laboratory of Molecular Epigenetics of MOE and Institute of Genetics & Cytology, Northeast Normal University, Changchun 130024, China

## Abstract

*Clivia miniata* is renowned for its evergreen and strap-like leaves, whereas its floral color and scent are lacking diversity. Here, anthocyanin, volatile terpene, and carotenoid metabolisms were integrally investigated in *C. miniata* flowers. The results showed that pelargonidins and lutein might cooperate to confer orange or yellow color to *C. miniata* flowers, but only a trace amount of (+)-limonene was detected. The expression levels of *CmF3′H* and *CmDFR* appeared to be responsible for the ratio of cyanidin and pelargonidin derivatives in *C. miniata*, and the low expression of *CmF3′H* was responsible for the lack of cyanidins in flowers. Moreover, the *CmF3′H* promoter could not be activated by CmMYBAs, suggesting that it was controlled by novel regulators. Only two CmTPSs were functional, with CmTPS2 responsible for (+)-limonene synthesis, contributing to the monotonous flower volatile terpenes of *C. miniata*. CmCCD1a and CmCCD1b were able to cleave carotenoids at the 5,6 (5′,6′), and 9,10 (9′,10′) positions to generate volatile apocarotenoids, whereas the substrates found in low-quantities or specific subcellular localizations of CmCCD1s might constrain volatile apocarotenoid release. Consequently, activating *F3′H* and introducing novel *F3′5′H* or versatile *TPS* may be effective ways to modify the floral color and scent, respectively. Alternatively, modifying the carotenoid flux or CCD1 localization might affect floral color and scent simultaneously. Taking these results together, the present study provides a preliminary deciphering of the genetic constraints underlying flower color and scent development, and proposes possible schemes for further genetic modification of ornamental traits in *C. miniata* and other plants.

## Introduction

The vigorous floriculture industry has brought considerable economic development and is expected to grow at a compound annual growth rate of >7% during the forecast period 2021–28 (DataM Intelligence; https://www.datamintelligence.com/research-report/floriculture-market). Flower crops with a diversity of ornamental traits, e.g. flower color and scent, are enjoyed by the consumer market. Generally, chlorophylls, flavonoids, betalaines, and carotenoids are well-known classes of natural pigments for coloration in plants, among which anthocyanins are the most important water-soluble flavonoids, endowing the flowers of most plants with red, purple, and blue coloration, whereas betalaines are mainly confined to Caryophyllales [1–4]. On the other hand, hydrophobic carotenoids are responsible for colors ranging from yellow through orange to red and could be co-accumulated with anthocyanins [5]. A number of compounds with different origins, terpenes, benzenoids/phenylpropanoids, and fatty acid derivatives, especially terpenes with relatively small molecular weight (monoterpenes and sesquiterpenes), are volatile organic compounds responsible for the floral scent [6, 7]. Those volatile organic compounds with relatively low odor thresholds perceived by people might play pivotal roles in flower fragrance [8–10]. Moreover, compounds responsible for floral color or scent are of great nutritional, therapeutic, or cosmetic value, and attract much attention from both botanist and citizen. For the plants, flower color and scent, together with other floral traits such as shape, size, and nectar, have long been identified as pollination signals that have co-evolved between plants and their pollinators. Although metabolites responsible for flower color or scent have different origins, both traits are found to have co-varied during the evolution and domestication of flowering plants [11–13].

It is widely accepted that anthocyanins are end-products of the flavonoid pathway. The basic skeleton of anthocyanins consists of three aromatic rings and is initialized by chalcone synthase (CHS) and chalcone isomerase (CHI), converting one molecule of ρ-coumaroyl-CoA and three molecules of malonyl-CoA into naringenin chalcone, and then naringenin consecutively. Flavonoid 3-hydroxylase (F3H) subsequently catalyzes the oxidation of the central ring to generate dihydrokaempferol (DHK), which can be further hydroxylated by flavonoid 3′-hydroxylase (F3′H) and flavonoid 3′5′ hydroxylase (F3′5′H), yielding dihydroquercetin (DHQ) and dihydromyricetin (DHM), respectively. The dihydroflavonols (DHK, DHQ, and DHM) can be further converted successively by dihydroflavonol reductase (DFR), leucoanthocyanidin oxidase (LDOX, also called ANS, anthocyanidin synthase) and 3-glucosyl transferase (3GT) to yield pelargonidin-, cyanidin-, and delphinidin-derived anthocyanins, accordingly. Alternatively, F3′H and F3′5′H could also introduce hydroxyl groups to naringenin at the 3′- or the 3′- and 5′-positions to generate 3′,4′-hydroxylated eriodictyol or 3′,4′,5′-hydroxylated pentahydroxyflavanone, respectively. Subsequently, eriodictyol and pentahydroxyflavanone could be further catalyzed by F3H to produce DHQ and DHM, respectively. Furthermore, other flavonoids share partial biosynthetic pathways with anthocyanins. For instance, flavonol synthase (FLS) can compete for dihydroflavonols to generate flavonols, while leucoanthocyanidin reductase (LAR) or anthocyanidin reductase (ANR) can redirect the anthocyanin flux to proanthocyanidin (PA) synthesis by reduction of leucoanthocyanidins or anthocyanidins [3, 14]. As stated above, F3H, F3′H, F3′5′H, and DFR are the key enzymes acting at the pathway bifurcations, whose expressions or enzymatic properties are responsible for specific anthocyanin biosynthesis. Moreover, a series of studies revealed that the expressions of anthocyanin biosynthetic genes in specific tissues or developmental stages are regulated transcriptionally by transcription factors [15, 16]. The most well-known regulator is the classical MBW complex composed of MYB-bHLH-WD40 proteins, such as AtPAP1-AtTT8-AtTTG1, PhAN2-PhAN2-PhAN11, and FhPAP1-FhTT8L-FhTTG1 [3,17–20]. In the complex, bHLH proteins may have overlapping regulatory targets, while WD40 proteins are expressed more or less ubiquitously and have debatable roles in transcriptional activation [21, 22]. MYB proteins are the most conspicuous components to activate discrete subsets of genes and therefore determine target gene expression patterns [15, 16].

As stated above, the volatile monoterpenes and sesquiterpenes are among the most abundant components of floral scent. Generally, the methylerythritol phosphate (MEP) pathway, confined to plastids, and the mevalonic acid (MVA) pathway, operating in the cytosol, are two well-characterized pathways that generate the 5-carbon precursors isopentenyl diphosphate (IPP) and its allylic isomer dimethylallyl diphosphate (DMAPP) [23]. A series of enzymes participating in MVA and MEP pathways have been identified, among which the rate-limiting enzymes are 3-hydroxy-3-methylglutaryl-coenzyme A (HMG-CoA) reductase (HMGR), 1-deoxy-d-xylulose 5-phosphate synthase (DXS), and 1-deoxy-d-xylulose 5-phosphate reductoisomerase (DXR) [23–25]. Different molecules of IPP and DMAPP can be further joined together to create the direct terpene precursors composed of geranyl diphosphate (GPP), farnesyl diphosphate (FPP), and neryl diphosphate (NPP), etc. Subsequently, the versatile terpene synthases (TPSs) convert these precursors into diverse terpenes. In particular, TPS could not only utilize multiple substrates to generate multi-products but also catalyze a single kind of substrate into various terpenes with one or two kinds of terpenes as main products and others as by-products [26–29]. Consequently, the catalytic capacity, plasticity, or low substrate-specificity of TPSs largely determines the abundance of volatile terpenes and affects floral scent.

In addition to the volatile terpenes, the MEP pathway also gives rise to other kinds of terpenes, such as diterpenes and tetraterpenes [5, 30, 31]. For example, the aforementioned C5 substrates IPP and DMAPP can be condensed into (C20) geranylgeranyl diphosphate (GGPP), which can then condense in a ‘head-to-head’ or ‘tail-to-tail’ fashion to form phytoene, the precursor of carotenoid. Phytoene can be sequentially transformed by phytoene desaturase (PDS), 15-*cis*-ζ-carotene isomerase (ZISO), ζ-carotene desaturase (ZDS), and carotenoid isomerase (CISO) to form the red lycopene, which can be further diverged into the yellow β-carotene and α-carotene by lycopene β-cyclase (LCYB) and lycopene ε-cyclase (LCYE), respectively. Afterwards, α-carotene can be catalyzed by carotene ε-monooxygenase (LUT) to form the yellow lutein with antioxidant effects. β-Carotene can be utilized by carotenoid β-hydroxylase (BCH) to generate the yellow zeaxanthin, an isomer of lutein. Concurrently, the mentioned carotenoids mentioned above can also be cleaved by carotenoid cleavage dioxygenases (CCDs) to produce myriad apocarotenoids in plants [32, 33]. The characterization of the first CCD could be traced back to the mutant *viviparous14* (VP14) in 1997 [34]. Later, Tan *et al*. identified nine *Arabidopsis* CCDs and further classified them into two groups including CCD1, CCD4, CCD7, CCD8, and 9-*cis*-epoxy-carotenoid dioxygenase (NCED)-based NCED2, NCED3, NCED5, NCED6, and NCED9, which laid the foundation of the subsequent CCD classification [35]. Recently, novel CCDs have been successively characterized, such as CCD2 from Iridaceae and CCD10 from maize (*Zea mays*) [36, 37]. Generally, all the NCEDs are involved in abscisic acid (ABA) formation, whereas CCD7 and CCD8 mainly participate in strigolactone biosynthesis [33, 38]. CCD1, CCD4, CCD2, and CCD10 can cleave diverse carotenoids at different double-bond sites to form various volatile apocarotenoids, such as β-ionone, pseudoionone, and 6-methyl-5-hepten-2-one (MHO), which affect not only the color but also the scent of plants [33, 38].

**Figure 1:**
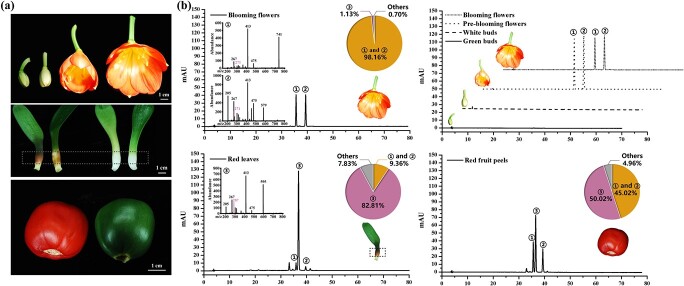
Anthocyanin accumulation profiles in floral developmental stages and different organs of *C. miniata.*  **a** Flowers at developmental stages and leaves or fruits with different colors. The region of leaves in the dotted box was used for further HPLC, HPL–ESI–MS, and gene expression analysis. **b** Anthocyanin analysis of colored *C. miniata* organs by HPLC or HPLC–ESI–MS. Three representative peaks detected by HPLC in *C. miniata* were numbered 1, 2, and 3. The peaks were further analyzed by HPLC–ESI–MS and represented by vertical drop lines in each panel. Peaks 1 and 2 were characterized as pelargonidin derivatives with the typical mass charge ratio of 271, while peak 3 was identified as cyanidin-based anthocyanins as a mass charge ratio of 287 was detected. The pie charts represent the relative contents of anthocyanidin derivatives detected in the different organs.

Some plant species can only accumulate or release limited sorts of natural pigments or volatile compounds, and thus exhibit monotonous color or a specific scent, which restrains their horticultural or nutritional values. *Clivia miniata* is a herbaceous perennial ornamental plant in the African genus *Clivia* (Amaryllidaceae). It was reported that species of *Clivia* had a late-acting self-incompatibility system, and largely depended on pollinators for seed production [39]. For example, the evolutionarily late-derived species *C. miniata* was predominantly pollinated by butterflies in the wild [39], whereas the molecular or biochemical basis for the visual or olfactory signals attracting pollinators are less investigated. Regardless of the pharmaceutical values reported [40], *C. miniata* has great economic and ornamental values as it is often deemed to be a symbol of gentlemen of honor and nobility. However, several limitations have greatly restrained the ornamental values of *C. miniata*, such as the relatively monotonous floral color and scent. Generally, the flowers of *C. miniata* appear orange or red to humans and emit a relatively simple range of volatiles mainly dominated by benzenoids/phenylpropanoids such as benzaldehyde, benzyl alcohol, and benzyl benzoate, and less volatile terpenes have been detected [39]. Genetic modification is becoming an effective way to overcome the limitations and introduce novel color or scent to plants [41–43]. However, comprehensive molecular investigation of the mechanism or drawbacks underlying the pigment or scent formation of *C. miniata* is a prerequisite for further genetic modification. In present study, the floral color and scent of *C. miniata* were thoroughly investigated mainly from the perspectives of anthocyanins, volatile terpenes, and carotenoids. We found that *C. miniata* could synthesize different kinds of anthocyanins in different tissues or organs. The expression of *F3′H* or introduction of novel F3′5′H may have a large effect on the floral color of *C. miniata*. As for volatile terpene biosynthesis, the main volatile terpene detected was (+)-limonene, which was catalyzed by CmTPS2. Activating more CmTPSs or introducing versatile TPSs from other plants may be effective ways to modify the floral scent. In addition, carotenoid metabolism could affect floral color and scent simultaneously. Lutein was the most abundant carotenoid accumulated and conferred a large portion of the yellow color on the flower. Concurrently, CmCCD1s were able to cleave the carotenoids at 5,6 (5′,6′) and 9,10 (9′,10′) positions, and may be targets to change floral color and improve the floral scent of *C. miniata* simultaneously. Taken together, the results of the present study not only provide preliminary deciphering of the possible mechanisms underlying floral color and scent formation of *C. miniata*, but also lay the foundations for further *Clivia* breeding by molecular design.

## Results

### Anthocyanins accumulated in *C. miniata*

For a preliminary investigation of the pigments in colored tissues or organs of *C. miniata*, we firstly separated anthocyanins and carotenoids, which helped visualize the differential accumulation of the anthocyanins and carotenoids in these samples (Supplementary Data Fig. S1). Subsequently, the anthocyanins were analyzed in detail by HPLC or HPLC–ESI–MS. Typically, the flower of *C. miniata* is pigmented with orange colors of different intensities. Flower development was divided into four stages: green bud stage, white bud stage, flower bud just after anthesis stage (pre-blooming), and fully opened flower stage (blooming) (Fig. 1a). Two typical peaks were detected in flowers and characterized as pelargonidin derivatives, which accounted for >98% of the total anthocyanins in this organ. As for flower development, the anthocyanin content was highest in flower buds just after anthesis (Fig. 1b). Moreover, the leaf stalk base of young seedlings could be colored when exposed to high light (Fig. 1a), and an obvious peak identified as cyanidin-based anthocyanins accounted for >82%, while the pelargonidin derivatives were <10% (Fig. 1b). The fruit peel of *C. miniata* also changed its color from green to red when fully ripened (Fig. 1a), and the content of cyanidin derivatives in red fruit peels was nearly the same as the content of pelargonidin derivatives. No anthocyanin was detected in non-colored leaf-stalk bases or green fruit peels.

### Expression analysis of anthocyanin biosynthetic genes in *C. miniata*

**Figure 2:**
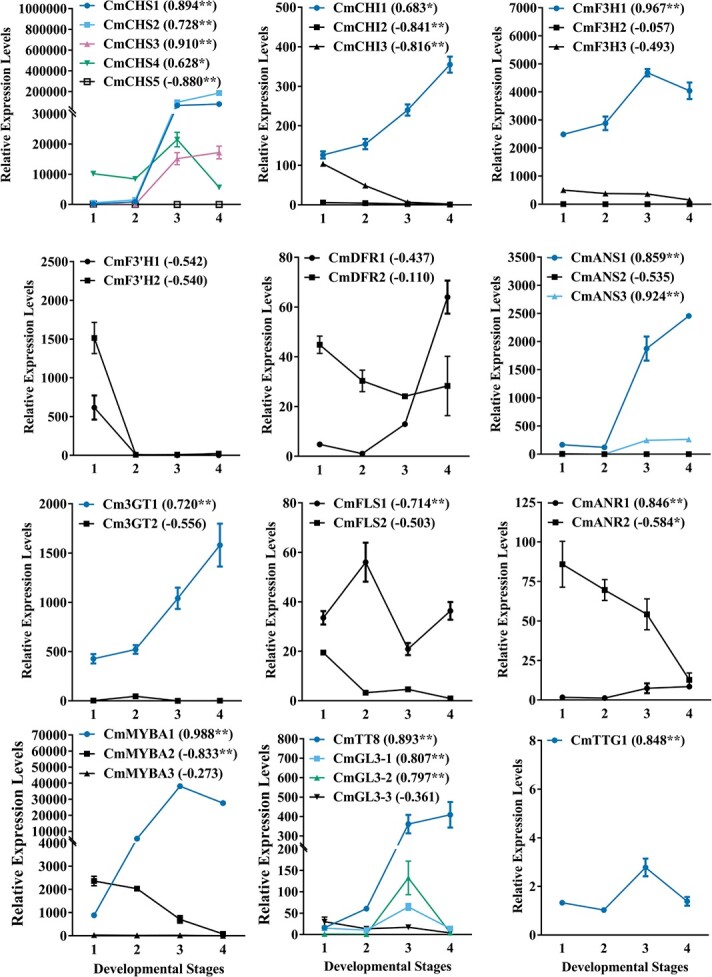
Expression profiles of flavonoid-related genes in flowers at different developmental stages. Data are mean ± standard deviation of at least three replicates. Transcripts were normalized by *Actin* and *ubiquitin.* Numbers under the *x*-axis indicate the four developmental stages of flowers and numbers in brackets are Pearson’s coefficients between gene expression and anthocyanin accumulation. Expression patterns of genes with positive coefficients are marked with colored lines. ^*^P < .05, ^**^P < .01, ^***^P < .001.

The anthocyanin biosynthetic genes were mined from a transcriptomic database of *C. miniata* that contained transcripts from flowers, leaves, and fruit peels (Supplementary Data Table S1). The expression patterns of these genes were firstly analyzed in flowers by qRT–PCR. Pearson’s correlation analysis showed that the expressions of *CmCHS1*, *CmCHS2*, *CmCHS3*, *CmCHS4*, *CmCHI1*, *CmF3H1*, *CmANS1*, *CmANS3*, and *Cm3GT1* were significantly correlated with total anthocyanin accumulation (Fig. 2). Interestingly, no candidate *CmF3′5′H* genes were found and the transcripts of *CmF3′H1* and *CmF3′H2* decreased sharply with flower development, which coincided well with the finding that tiny contents of cyanidin derivatives were detected in this organ. Moreover, other flavonoid biosynthetic genes, such as flavonol-related *FLS* and proanthocyanidin related-*ANR* (no LAR candidates were mined), were also analyzed (Supplementary Data Table S1) and generally showed negative correlations with anthocyanin biosynthesis (Fig. 2). As the canonical MBW components, three MYB activators, four bHLH factors, and one WD40 protein were mined (Supplementary Data Table S1) and most of the transcripts showed significant positive correlations with total anthocyanin accumulation (Fig. 2).

**Figure 3:**
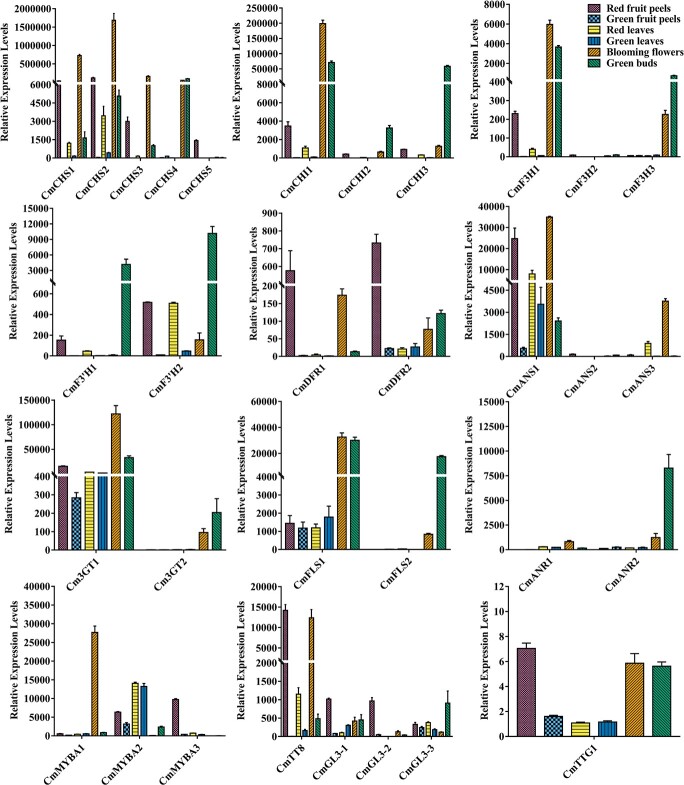
Expression profiles of flavonoid-related genes in colored tissues or organs of *C. miniata*. The tissues and organs are illustrated in Fig. 1. Data are mean ± standard deviation of at least three replicates. Transcripts were normalized by *Actin* and *ubiquitin.*

The transcripts of anthocyanin-related genes, as well as *CmFLS* and *CmANR*, were further analyzed among different tissues or organs of *C. miniata.* Generally, most transcripts showed higher expression levels in red-colored tissues or organs (Fig. 3). As *F3H*, *F3′H* and *DFR* were located in the branching positions of anthocyanin biosynthesis mentioned above, their expression specificities deserved special attention to unveil the molecular mechanisms underlying spatial anthocyanin biosynthesis in *C. miniata*. As shown in Fig. 3, *CmF3H1* showed much higher expression levels than *CmF3H2* or *CmF3H3* in all the tests and might be the pivotal gene responsible for anthocyanin biosynthesis. Though *CmF3′H2* was more highly expressed than *CmF3′H1*, they shared a similar expression pattern in the different tissues or organs studied. However, only trace transcripts of *CmF3′H1* or *CmF3′H2* were expressed in blooming flowers, which might explain why cyanidin-based anthocyanins could hardly be detected in flowers. As for *DFR* genes, *CmDFR1* had expression levels similar to those of *CmDFR2* in red fruit peels and blooming flowers. However, *CmDFR2* transcripts were nearly 20 times higher than *CmDFR1* in red leaves, which mainly accumulated cyanidin-based anthocyanins. The results suggested that the expression specificity of *CmDFR2* might also play a part in specific anthocyanin biosynthesis in *C. miniata.*

As for the MBW components, TT8-clade bHLH factor *CmTT8* had higher expression levels when compared with the three GL3-clade factors in all the tissues or organs studied. It is worth mentioning that the three potential MYB activators were also differently expressed in these tissues or organs. *CmMYBA1* and *CmMYBA2* were mainly expressed in flowers and leaves, respectively, while *CmMYBA3* tended to function in red fruit peels (Fig. 3). Whether the different expressions of these MYB activators regulated different structural gene expressions and further resulted in specific anthocyanin biosynthesis remains to be analyzed further, however.

### CmF3H1 could convert naringenin and eriodictyol into dihydrokaempferol and dihydroquercetin, respectively

As *CmF3H1* was most abundantly expressed among the three *F3H*s, it was subjected to further analysis. The 1098-bp open reading frame (ORF) encoding 365 amino acids was cloned. Amino acid sequence alignment revealed the conserved Fe^2+^-binding sites, oxoglutarate-binding sites, and 2-oxoglutarate-Fe (II) oxygenase domain, indicating CmF3H1 had potential biological functions as a member of the 2-ODD (2-oxoglutarate-dependent dioxygenase) superfamily (Supplementary Data Fig. S2a). Phylogenetic analysis including F3H, LDOX, and FLS, members of the 2-ODD superfamily indicated that CmF3H1 was more closely related to FhF3H from *Freesia hybrida* (Supplementary Data Fig. S2b), further implying its potential roles in the biosynthesis of anthocyanins.

**Figure 4:**
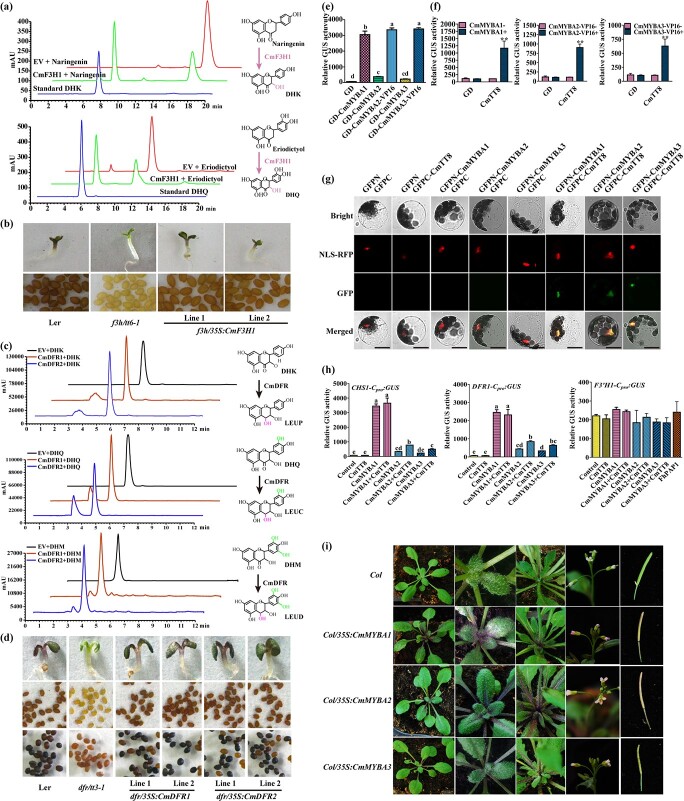
Functional characterization of anthocyanin-related genes in *C. miniata*. **a**  *In vitro* enzyme assay of CmF3H1 on naringenin and eriodictyol. **b** Functional complementation of *Arabidopsis f3h* mutant (*tt6–1*)*.*  **c**  *In vitro* enzyme assay of CmDFRs on DHK, DHQ, and DHM. **d** Phenotypes of wild-type (Ler, Landsberg), *dfr* mutant (*tt3–1*), and T2 transgenic lines of *CmDFRs*. The black seeds were stained by dimethylaminocinnamaldehyde (DMACA). **e** Transactivation capacities of CmMYBA1, CmMYBA2, CmMYBA3, and their modified versions. The strong viral activation domain VP16 was fused to the C-terminus of CmMYBA2 and CmMYBA3 to construct CmMYBA2-VP16 and CmMYBA3-VP16, respectively. **f** Protein–protein interactions between CmMYBAs and CmTT8 detected by Gal4-based transient protoplast assay. **g** Protein–protein interactions between CmMYBAs and CmTT8 detected by BiFC assay. **h** Assays of activation of *CHS1-C_pro_*, *DFR1-C_pro_*, and *F3′H-C_pro_* by the MYB-bHLH complex using *Arabidopsis* protoplasts isolated from the *tt8gl3egl3* triple mutant. **i** Phenotypes of wild-type and transgenic *Arabidopsis* overexpressing *CmMYBA*s. One-way ANOVA was carried out to compare statistical differences in (**e**) and (**h**) (Duncan, *P* < .05). The *t*-test was used to analyze significant differences in (**f**). ^**^P < .01. Data are the mean ± standard deviation of three replicates.

To investigate the enzymatic activity of CmF3H1 *in vitro*, the recombinant protein expressed in prokaryotic *Escherichia coli* BL21 (DE3) was purified with a nickel column and further incubated with naringenin or eriodictyol. Consequently, both naringenin and eriodictyol could be catalyzed by CmF3H1 protein to generate DHK and DHQ, respectively, demonstrating its role in flavonoid biosynthesis (Fig. 4a). To emphasize the role of CmF3H1 *in vivo*, the *Arabidopsis tt6-1* mutant (NASC stock number NW87), lacking flavanone 3-hydroxylase, was selected to investigate the functionality of CmF3H1. The results showed that transgenic seedlings displayed red cotyledons and brown seeds similar to those of wild-type *Arabidopsis*, whereas cotyledons and seeds of the mutant were green and transparent, respectively (Fig. 4b). Based on the results mentioned above, CmF3H1 offered the possibility that flavanone could be hydroxylated at the third position in two different manners during anthocyanin biosynthesis in *C. miniata.*

### No significant difference was observed between CmDFR1 and CmDFR2 in catalyzing dihydroflavonols

As the enzyme properties of DFR proteins were also critical for specific anthocyanin biosynthesis in plants, CmDFR1 and CmDFR2, which were most likely to be *bona fide* DFRs in the NADPH-dependent reductase superfamily, were cloned and they turned out to share 68 and 65% identities with *Arabidopsis* DFR, respectively (Supplementary Data Table S1). Amino acid sequence alignment with DFRs and cynnamoyl CoA reductase (CCRs), members of the NADPH-dependent reductase superfamily, from other plants revealed the putative NADP-binding region and substrate-binding region (Supplementary Data Fig. S3a). A phylogenetic tree containing other NADPH-dependent reductases showed that CmDFR1 and CmDFR2 were clustered within the DFR clade as expected (Supplementary Data Fig. S3b).

To further investigate whether the substrate specificities of CmDFRs resulted in the imbalanced accumulation of different anthocyanins in *C. miniata*, enzymatic properties of CmDFR1 and CmDFR2 with DHK, DHQ, and DHM as substrates were tested in the presence of NADPH. As shown in Fig. 4c, both CmDFR1 and CmDFR2 could convert DHK, DHQ, and DHM into leucopelargonidin (LEUP), leucocyanidin (LEUC), and leucodelphinidin (LEUD), respectively. In order to further investigate their roles in flavonoid biosynthesis *in planta*, *CmDFR1* and *CmDFR2* were constitutively expressed in the *Arabidopsis tt3–1* mutant (NASC stock number NW84), which lacked pigments in the cotyledon, hypocotyl, or seed coat because of *AtDFR* deficiency. As expected, either CmDFR1 or CmDFR2 complemented the mutant with pigmentation of germinating seedling cotyledons and brown seeds to levels comparable to those in wild-type *Arabidopsis* (Fig. 4d). Taken together, these results indicate that substrate specificities of CmDFR1 and CmDFR2 might play lesser roles in determining specific anthocyanin biosynthesis in *C. miniata.*

### CmMYBA-CmTT8 complexes could regulate early and late anthocyanin biosynthetic genes except *CmF3′H*

Three MYB regulators were identified (Supplementary Data Table S1) and confirmed by sequence alignment (Supplementary Data Fig. S4a) as well as phylogenetic analysis (Supplementary Data Fig. S4b). To better define these CmMYBs, a Gal4-based transient protoplast assay was performed [19, 44–47]. *CmMYBA1*, *CmMYBA2*, and *CmMYBA3* were fused to the GD tag and co-transfected with the *GUS* reporter gene driven by the Gal4 promoter. The results showed that CmMYBAs were transactivators, as significant GUS activities were detected when compared with the GD control (Fig. 4e). Moreover, the transactivation capacity of CmMYBA1 was much stronger than those of CmMYBA2 and CmMYBA3. Then, the strong activation domain VP16 (viral activation domain) was fused to CmMYBA2 and CmMYBA3 to strikingly heighten their transactivation capacities (Fig. 4e). To verify the potential interactions between *C. miniata* bHLH factor and CmMYBAs, CmTT8 was isolated and fused to the GD tag to construct GD-CmTT8 vector, which was further co-transfected with 35s promoted CmMYBAs, and the reporter construct containing Gal4 promoted GUS (Fig. 4f). If any of the CmMYBAs could interact with CmTT8, significant GUS activities would be detected as the MYB activator would be dragged by GD-CmTT8 to the promoter of the *GUS* reporter gene. The results showed that CmMYBA1, CmMYBA2, and CmMYBA3 could interact with CmTT8 to form MYB-bHLH complexes (Fig. 4f). In addition, the bimolecular fluorescence complementation (BiFC) assay re-confirmed that all three CmMYBA proteins could interact with CmTT8 in the nucleus (Fig. 4g).

To better understand the regulation of anthocyanin biosynthetic genes by the *Clivia* MYB-bHLH complexes, *CmCHS1* and *CmDFR1* were selected as representatives of early and late anthocyanin biosynthetic genes. Subsequently, the −973 and −262 bp upstream of the initiation codon ATG were cloned as promoters of *CmCHS1* and *CmDFR1*, respectively*.* The predicted promoters were subcloned to promote the *GUS* reporter gene, then co-transfected with either CmMYBAs or CmTT8 into plant protoplasts. As shown in Fig. 4h, both *CmCHS1* and *CmDFR1* could be dramatically activated by CmMYBA alone or together with CmTT8. In more detail, CmMYBA1 had much stronger effects on anthocyanin biosynthetic genes than CmMYBA2 or CmMYBA3. As the expression of *CmF3′H* was consistent with spatial anthocyanin biosynthesis in *C. miniata*, whether it could be activated by the *Clivia* regulators was also determined. In addition, the powerful FhPAP1 earlier characterized from *F. hybrida* was included as control [13]*.* However, none of the regulators could distinctly activate the *CmF3′H1* promoter, even in the presence of CmTT8 (Fig. 4h), suggesting that the *CmF3′H* promoter might be differentially regulated in *C. miniata* independently of the canonical MBW complex.

The functions of the CmMYBAs in the whole plant were further checked in *Arabidopsis*. Expression of *CmMYBA*s in *Arabidopsis* resulted in increased pigmentations in different tissues or organs, even flowers, by promoting anthocyanin biosynthetic genes, including *AtF3′H* (Fig. 4i; Supplementary Data Fig. S5a and b). In addition, GD-AtTT8 evidently activated *GUS* expression only in the presence of CmMYBAs, corroborating the idea that CmMYBAs interacted with AtTT8 in *Arabidopsis* (Supplementary Data Fig. S5c). Interestingly, CmMYBAs and CmTT8 could not activate the *CmF3′H* promoter, whereas overexpression of *CmMYBA*s in *Arabidopsis* activated *AtF3′H*. We further transiently transformed *CmMYBA*s and *CmTT8* into *Arabidopsis* protoplasts either alone or concurrently to detect their effects on *AtF3′H*. The results showed that CmMYBA1, CmMYBA2, and CmMYBA3 significantly activated *AtF3′H* and that CmTT8 could strengthen the activation efficiency (Supplementary Data Fig. S5d). In conclusion, the *CmF3′H* promoter might be somewhat peculiar because it could not be activated by the canonical MYB-bHLH complex.

**Figure 5:**
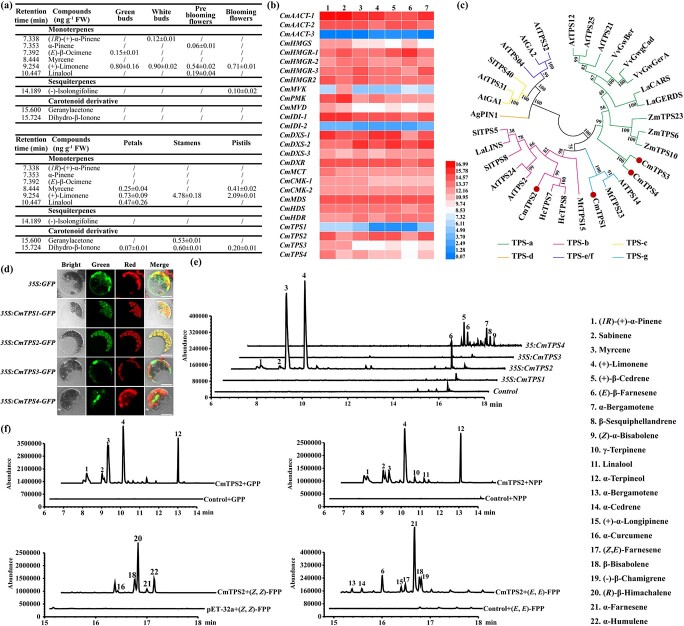
Functional characterization of volatile terpene-related *TPSs* in *C. miniata*. **a** Volatile terpenes and apocarotenoids detected in *C. miniata* flowers. The slash (/) indicates tentatively detected or undetected. Data are mean ± standard deviation of three replicates relative to (+)-limonene. **b** Relative transcript expression levels involved in terpene biosynthesis in *C. miniata*. Transcripts were normalized by *Actin* and *ubiquitin*, and compared with the lowest expression level of the specific gene in a specific stage or tissue. All data were calculated as log_2_. Red and blue boxes indicated high and low expression levels, respectively. 1–4 represent the four developmental stages of flowers; 5–7 indicate petals, stamens, and pistils, respectively. **c** Phylogenetic analysis of *C. miniata* TPS sequences. The TPS sequences were processed by Clustal Omega and subjected to MEGA-X to construct the neighbor-joining tree. Bootstrap values are based on 1000 replicates. Different TPS clades are highlighted with differently colored lines. The following GenBank accession numbers were used: AtTPS2 (NP_193406.3), AtTPS04 (Q93YV0.1), AtTPS12 (Q9T0J9.2), AtTPS14 (NP_001185286.1), AtTPS21 (NP_001190374.1), AtTPS24 (NP_189209.2), AtTPS25 (NP_001325487.1), AtTPS31 (NP_192187.1), AtTPS32 (NP_178064.1), AtGA1 (Q38802.1), AtGA2 (NP_178064.1), SlTPS5 (NP_001233805.1), SlTPS8 (XP_004231365.1), SlTPS40 (NP_001234008.2), AgPIN1 (O24475.1), MtTPS15 (XP_003621227.1), MtTPS23 (XP_003619707.1), ZmTPS6 (NP_001105674.1), ZmTPS10 (NP_001105850.1), ZmTPS23 (ABY79213.1), VvGwGerA(ADR66821.1), VvGwBer (ADR74195.2), VvGwgCad (ADR74199.1), HcTPS7 (AHJ57305.1), HcTPS8 (AGY49283.1), LaCARS (AGL98419.1), LaGERDS (AGL98420.1), LaLINS(Q2XSC5.1). **d** Subcellular localizations of CmTPS1–4 in *Arabidopsis* protoplasts. **e**  *In vivo* transient expressions of *CmTPS1–4* in *N. benthamiana* leaves. **f**  *In vitro* enzymatic analysis of CmTPS2 using four acyclic prenyl diphosphate substrates: GPP, NPP, (*E,E*)-FPP, or (*Z,Z*)-FPP. Numbers in (**e**) and (**f**) represent volatile terpenes listed in the key at bottom right.

### CmTPS partially accounts for monotonous floral volatile terpenes

Consistent with an earlier study [39], benzenoids/phenylpropanoids such as benzaldehyde and benzyl alcohol were also detected in our study and confirmed to constitute the predominant volatiles in *C. miniata* flowers (Supplementary Data Fig. S6). (+)-Limonene was the only volatile terpene constitutively detected in floral tissues or organs, whereas several other terpenes, such as linalool and myrcene, were measured in specific samples (Fig. 5a). The structural genes involved in the terpene biosynthetic pathway were tentatively mined from the constructed transcriptomic database (Supplementary Data Table S1) and their relative expression levels were evaluated by qRT–PCR (Fig. 5b). Irrespective of the enzymatic efficiencies, the gene expression levels implied their encoded enzymes might yield enough GPP, FPP, or NPP, which could be directly converted into volatile terpenes by TPSs. Consequently, the versatile TPSs might have mainly resulted in the structurally diverse terpenes. However, only four *TPS* genes were obtained (Supplementary Data Table S1). Phylogenetic analysis indicated that CmTPS2 fell into the TPS-b subclade responsible for monoterpene synthesis, and CmTPS3 and CmTPS4 belonged to the sesquiterpene-related TPS-a subclade, whereas CmTPS1 might function as monoterpene or sesquiterpene synthase as it clustered into the TPS-g family (Fig. 5c). Amino acid sequence alignment revealed the conserved DDXX(D/E) and (N,D)DXX(S,T,G) XXXE (NSE/DTE) that functioned in binding Mg^2+^ or Mn^2+^ cofactors, as well as terpene cyclization-related RR(X)_8_W (Supplementary Data Fig. S7). Subcellular localization observation showed that CmTPS3 and CmTPS4 were cytosol-localized proteins whereas CmTPS1 and CmTPS2 were confined to plastids (Fig. 5d), further implying the different roles among the CmTPSs. To characterize the functionality of these four CmTPSs, they were transiently expressed in *Nicotiana benthamiana* leaves, which were subjected to volatile terpene detection. Interestingly, no volatile terpenes were detected in tobacco leaves expressing *CmTPS1* or *CmTPS3*, whereas leaves expressing *CmTPS2* produced a large amount of (+)-limonene and myrcene (Fig. 5e; Supplementary Data Table S2). In comparison, CmTPS4 could result in a series of terpenes, such as (+)-β-cedrene, α-bergamotene and (*E*)-β-farnesene, but in relatively low amounts (Fig. 5e, Supplementary Data Table S2). The *in vivo* results obtained with CmTPS2 coincided well with the major terpenes detected in *Clivia* flowers. Moreover, enzymatic assays revealed that the recombinant CmTPS2 could convert GPP, NPP, (*E*,*E*)-FPP, or (*Z*,*Z*)-FPP into a wide spectrum of products, suggesting that CmTPS2 was generally a versatile enzyme (Fig. 5f; Supplementary Data Table S3). On the whole, it may be that CmTPS2 results in constitutive (+)-limonene release in *Clivia* flowers, and that the smaller number of copies, low transcripts, and inactive enzymatic properties of CmTPSs have largely restrained the floral volatile terpenes.

**Figure 6:**
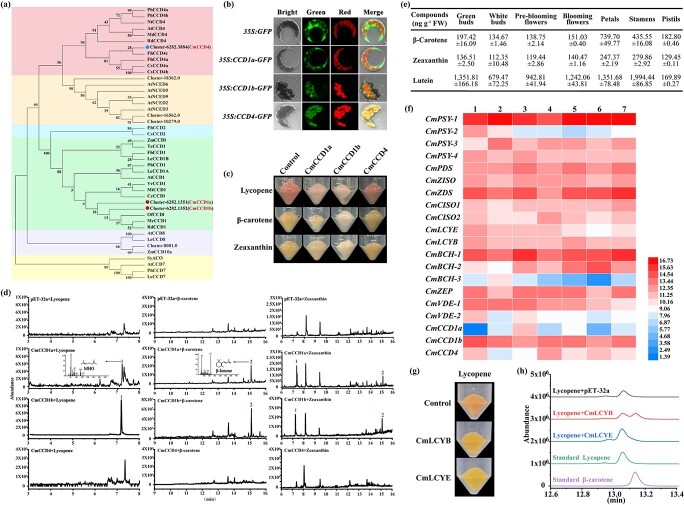
Functional characterization of CmCCDs and carotenoid biosynthesis in *C. miniata*. **a** Phylogenetic analysis of *C. miniata* CCD sequences. The CCD sequences were processed by Clustal Omega and subjected to MEGA-X to construct the neighbor-joining tree. Bootstrap values were based on 1000 replicates. The following GenBank accession numbers were used: TeCCD1 (AOS85165.1), NtCCD4 (NP_001313051.1), AtCCD1 (CAA06712.1), AtCCD4 (O49675.1), AtCCD7 (Q7XJM2.1), AtCCD8 (Q8VY26.1), AtNCED2 (O49505.1), AtNCED3 (Q9LRR7.1), AtNCED5 (Q9C6Z1.1), AtNCED6 (Q9M9F5.1), AtNCED9 (Q9M9F5.1), CsCCD2 (ACD62475.1), CsCCD4a (QEY87979.1), CsCCD4b (QEY87979.1), PhCCD1 (AAT68189.1), PhCCD7 (ACY01408.1), OfCCDl (AXQ60417.1), VvCCD1 (AXQ60417.1), LeCCD1A (NP_001234542.1), LeCCD1B (NP_001233838.1), LeCCD7 (NP_001234433.1), LeCCD8 (AEH96363.1), MtCCD1 (CAR57918.1), CcCCDl (ABA43900.1), ZmCCDl (CAR57918.1), McCCD1 (AFU91489.1), RdCCD1 (ABY47994.1), RdCCD4 (ABY60886.1), MdCCD4 (ABY47995.1), PhCCD4a (QBC36238.1), PhCCD4b (QEH92024.1), ZmCCD10a (GRMZM2G164967_T01), SyACO (P74334). **b** Subcellular localizations of CmCCDs in *Arabidopsis* protoplasts. **c** Functional characterization of CCDs in *E. coli* cells accumulating lycopene, β-carotene, or zeaxanthin. **d** GC–MS analysis of the headspace of the *E. coli* cells accumulating lycopene, β-carotene, or zeaxanthin and CmCCDs. **e** Relative contents of carotenoids detected in *C. miniata* developed flowers or floral organs. Data are mean ± standard deviation of three replicates relative to lutein. **f** Relative transcripts of genes involved in carotenoid biosynthesis of *C. miniata*. The transcripts were normalized by *Actin* and *ubiquitin*, and compared with the lowest expression level of the specific gene in specific stages or tissues. All the data were calculated as log_2_. Red and blue boxes indicate high and low expression levels, respectively. 1–4 represent the four developmental stages of flowers; 5–7 indicate petals, stamens, and pistils, respectively. **g** decolorations of *E. coli* cells accumulating lycopene upon expression of CmLCYB or CmLCYE. **h** HPLC analysis of lycopene-accumulating *E. coli* cells expressing empty vector, CmLCYB, or CmLCYE.

### CmCCDs may be targets to change floral color and improve the floral scent of *C. miniata*

As stated in the Introduction, the plastidic MEP-derived IPP and DMAPP could be transformed into yellow- or orange-colored carotenoids, which would be further cleaved by CCDs to form a series of apocarotenoids affecting floral scent. Interestingly, we also detected the apocarotenoid dihydro-β-ionone in the floral scent bouquet (Fig. 5a). To further decipher the possible mechanism, three candidate CmCCDs were isolated and tentatively named CmCCD1a, CmCCD1b, and CmCCD4 (Fig. 6a). Amino acid sequence alignment revealed the four conserved histidine residues responsible for binding the Fe^2+^ cofactor (Supplementary Data Fig. S8). Subcellular localization assay showed that CmCCD1a and CmCCD1b were diffused in cytoplasm, whereas CmCCD4 was confined to plastids (Fig. 6b). When the coding sequences of *CmCCD1a*, *CmCCD1b*, and *CmCCD4* were expressed in engineered *E. coli* strains capable of synthesizing individual carotenoids with distinct colors, including lycopene, β-carotene, and zeaxanthin, evident decolorations were observed in the strains expressing *CmCCD1a* or *CmCCD1b* (Fig. 6c). In contrast, color intensities of *E. coli* pellets harboring *CmCCD4* were nearly consistent with the negative control (Fig. 6c). Solid-phase microextraction (SPME)–GC–MS analysis detected MHO in the cultures expressing lycopene, β-ionone in the cultures expressing β-carotene, and MHO and β-ionone in the cultures expressing zeaxanthin, respectively (Fig. 6d). However, no volatile was identified in the cultures expressing *CmCCD4* (Fig. 5D). Thus, CmCCD1a and CmCCD1b were able to cleave the above carotenoids at the 5,6 (5′,6′) and 9,10 (9′,10′) positions (Supplementary Data Fig. S9).

Though CmCCD1a and CmCCD1b could cleave carotenoids into MHO or β-ionone, we scarcely detected them in the volatiles from *Clivia* flowers. This phenomenon intrigued us to further speculate that the content of carotenoids captured as substrates by CmCCD1a or CmCCD1b or the expression levels of *CmCCDs* might be low in *Clivia* flowers. Accordingly, carotenoids were extracted from differently developed *Clivia* flowers or floral tissues and quantitatively analyzed by HPLC. Remarkably, only lutein, β-carotene and zeaxanthin were characterized, and lutein was the major one (Fig. 6e). qRT–PCR analysis was performed to mine the potential rate-limiting genes in the carotenoid pathway. Results showed that most of the structural genes had comparable expression levels (Fig. 6f), hinting that the enzymatic activities of the enzymes, especially lycopene cyclases at the bifurcation step, might play determinant roles. From the *Clivia* transcriptomic database, two genes that carried ORFs sharing high sequence similarities were cloned and named *CmLCYB* and *CmLCYE*, respectively (Supplementary Data Table S1). Amino acid sequence alignment revealed that the homologs shared conserved Rossmann fold domains, cyclase domains, predicted transmembrane helixes, and charged domains. Phylogenetic analysis indicated that CmLCYB and CmLCYE fell into different subclades with other functionally characterized LCYs (Supplementary Data Fig. S10). Subsequently, the bacterial pigment complementation system was used to determine the enzymatic activities of CmLCYB and CmLCYE. Consequently, the transformation of CmLCYB or CmLCYE into lycopene-accumulating *E. coli* resulted in the color change from pink to orange (Fig. 6g). HPLC analysis revealed an accumulation of the bicyclic β-carotene in bacteria co-transformed with CmLCYB, whereas a significant peak increase was observed to overlap with lycopene in bacterial extract co-expressing CmLCYE (Fig. 6h).

## Discussion

Phylogenetically, *C. miniata*, with unique trumpet-shaped flowers, is a derived species from the remaining pendulous flowered species in the *Clivia* genus [48, 49]. The reproductive biology of *Clivia* species revealed that the species in the genus have experienced pollinator transition from bird to butterfly, which was accompanied by shifts of pollination syndrome, e.g. flower shape, color, and scent. For example, flowers of the most recent derived Clivia species, *C. miniata* lacked green perianth tips but evolved a yellow throat at the base of the inner tepal, and emitted ~50-fold more scent than flowers from its relative *C. gardenii* [39]. Color cues together with scent would help butterflies in navigation for flowers. Moreover, *C. miniata* perhaps is the most widely cultivated species in the *Clivia* genus around the world and is commercially welcome in floriculture markets globally, especially in Australia, China, Belgium, Japan, New Zealand, and the USA [40]. Improving the traits of *C. miniata* has long been a concern of breeders and botanists. Among various horticultural traits, flower color and scent are usually the most important quality determinants affecting the ornamental merits and commercial values of an ornamental plant. Thus, understanding the mechanisms underlying flower color and scent development provides an important theoretical basis and premise for further cultivation and improvement of new varieties of *C. miniata*, considering the paucity of information regarding the metabolites of the genus.

It seems common that colors are limited in some ornamental plants in spite of the wide range of natural flower colors. Pursuing new cultivars with novel flower color, such as the blue chrysanthemum and rose, yellow cyclamen, and peony, has always been an important goal for breeders. Generally, pigment type and distribution, petal tissue structure, physical factors including temperature, light and water, and chemical factors, comprising pH value, mineral nutrients and plant hormones, etc. are factors affecting flower color, and among these anthocyanins are the most important flower color determinants [3, 50–53]. Firstly, functional loss of structural genes usually results in an evolutionary color transition from blue to red or colored to colorless between species, as loss of function is often easier than gain of function [54]. Secondly, the expression and enzymatic properties of F3′H, F3′5′H, and DFR determine branches of the anthocyanin pathway, further affecting flower coloration diversity [55, 56]. The enzymatic properties of enzymes at bifurcations of the flavonoid pathway may also influence flower coloration. Anthocyanins share most biosynthetic enzymes with other flavonoids, such as flavonol and proanthocyanidins. DFR competes with FLS for the common dihydroflavonols. Relative expression levels, substrate specialties, and catalytic efficiencies of DFR and FLS could alter metabolite flux to affect anthocyanin content [57]. Similarly, the competitive relationships of LDOX and LAR and of 3GT and ANR could also lead to color alteration [58–60]. In addition, >600 anthocyanins have been reported to date, whereas only six types of aglycones (pelargonidin, cyanidin, delphinidin, peonidin, petunidin, and malvidin) have been widely detected. Decoration enzymes such as glycosyl transferase, methyltransferase, and acyltransferase have transferred various decoration moieties to multiple positions of anthocyanidin molecules, leading to the diversification of anthocyanins [61, 62]. Consequently, mutation of these decoration enzymes would shift the color scheme of a plant. It is worth mentioning that all the above-mentioned genes are regulated by transcription factors, particularly the canonical MYB-bHLH-WD40 complex [3, 17–19]. The expression or regulatory properties of these regulators play vital roles in flower coloration [13, 57]. In the present study, pelargonidin-derived anthocyanins were mainly deposited in flowers of *C. miniata*, which might account for its monotonous colors (Fig. 1). The floral anthocyanin defects in this plant could be explained from the following aspects. Firstly, *F3′5′H* homologs failed to be identified from *C. miniata*, which corresponded well with the fact that no delphinidin-derived anthocyanins were detected in this plant (Figs 1–3; Supplementary Data Table S1). It is accepted that F3′5′H evolved from an F3′H precursor and a small number of amino acid exchanges would be enough for the change from 3′- to 3′,5′-hydroxylation activity [56, 63]. However, it is unclear whether the potential *CmF3′5′H* is lost in the *C. miniata* genome or silenced in the detected tissues or organs. Secondly, no substrate specificity difference was observed for CmDFR1 and CmDFR2 (Fig. 4c), further confirming that an expressed and functional F3′5′H was vital for *C. miniata* to synthesize delphinidin-derived anthocyanins in flowers. Thirdly, *CmF3′H*s were expressed at extremely low levels that blocked cyanidin derivatives and redirected DHK into the pelargonidin pathway as flower development proceeded (Figs 1–3). Interestingly, the promoter of *CmF3′H* seemed not to be controlled by CmMYBAs, or even the earlier-reported FhPAP1 with particularly high transactivation capacity [64] (Fig. 4h). Similar results were also observed in kiwifruit (*Actinidia* species) showing that *F3′H* and *F3′5′H* could not be activated by kiwifruit MYB10 or MYB110 [65], indicating that there might be other uncharacterized regulatory proteins directly responsible for *F3′H* or *F3′5′H* expression in some plant species. In conclusion, modifying the expressions or enzymatic properties of F3′H and F3′5′H may be a primary strategy to complement the drawbacks of flower color richness in *C. miniata*.

Compared with the in-depth and systematic molecular investigations of floral color, the characterization, biosynthesis, and regulation of floral scent has long lagged behind. So far, an increasing number of floral volatiles composed of terpenes, benzenoid aromatics, and fatty acid derivatives from angiosperms have been analyzed, such as the model *Arabidopsis* and tobacco [66, 67] and common cultivated horticultural plants (e.g. *Freesia* and rose) [27, 68]. As the mainstay among most floral volatile organic compounds, volatile terpenes showed species-specificity with the possibility of great variations among related species [26, 29]. It is widely accepted that floral terpenes function together with anthocyanins in the attraction of pollinators, dispersers and pest enemies, and biotic and abiotic resistances [6, 69]. Compared with anthocyanins, floral terpenes seem to be more sensitive to environmental stimuli, or specific terpenes could only be detected in specific development stages [70], which was also validated in *C. miniata* as fewer terpenes and terpenes that were present in smaller quantities were detected when normally cultivated (Fig. 5a). Overall, *C. miniata* was not in the same league as other familiar horticultural plants in the types and contents of volatile terpenes released from their flowers, which might be driven by TPSs. Generally, *TPS* genes varied in number, ranging from 1 to >100 in land plants [ 25, 71]. In *C. miniata*, only four *CmTPS* genes were mined to be expressed at varying levels (Fig. 5b and c). Considering that a specific TPS usually converts a specific kind of substrate into multiple products, and most TPS proteins could utilize several kinds of substrate, the four CmTPSs should have generated plenty of volatile terpenes. However, transient transformation assays indicated that not all the four CmTPSs were functional (Fig. 4e), and only C*mTPS2* was the major floral *TPS* gene, as its catalytic products mirrored the released flower scent. In conclusion, considering the ``less'' means the copy number of TPS is less in *C. miniata* than it in most plants and ``lower'' means the TPSs in *C. miniata* have lower catalytic properties of CmTPSs, introducing more versatile TPSs from other plants might be an effective way to modify the floral scent of *C. miniata*.

Considering the relatively independent metabolisms of anthocyanins and terpenes, it is hard to synchronously alter floral color and scent by modifying a single gene, notwithstanding several studies that have observed synergetic alterations [72–75]. Alternatively, carotenoid metabolism makes it possible, as yellow carotenoids could be cleaved into volatile apocarotenoids with low odor thresholds by CCDs [32, 38]. In this study, different quantities of lutein, β-carotene, and zeaxanthin were detected in *C. miniata*, which might confer the yellow color on the flower (Fig. 6e). Functional characterization of CmCCD1s indicated that lycopene, β-carotene, and zeaxanthin were all their substrates. However, zeaxanthin might not be the true substrate of CCD1s as MHO and β-ionone instead of 3-OH-β-ionone was detected (Fig. 6d). This might be interpreted as showing that CmCCD1 could efficiently cleave lycopene and β-carotene during zeaxanthin biosynthesis in the engineered bacteria. Consequently, the enzymatic properties and relatively higher expression level of *CmCCD1* indicated that the aromatic molecule MHO or β-ionone instead of dihydro-β-ionone should have been detected (Fig. 6d). Several possibilities should be considered. Firstly, β-ionone might be further reduced to dihydro-β-ionone by an uncharacterized enzyme in *C. miniata*. Secondly, the content of β-carotene was not enough to be cleaved by CmCCD1 as the carotenoid flux was mainly towards lutein (Fig. 6e). Tentatively, CmLCYE might have much higher catalytic efficiency than CmLCYB in utilizing lycopene, though there is a lack of adequate evidence. Furthermore, the cytosolic localization of CmCCD1 might constrain its catalytic property as carotenoids were deposited in plastids. Consequently, *CmCCD1* might be a candidate gene to improve the flower scent of *C. miniata* if it were localized in plastids.

**Figure 7:**
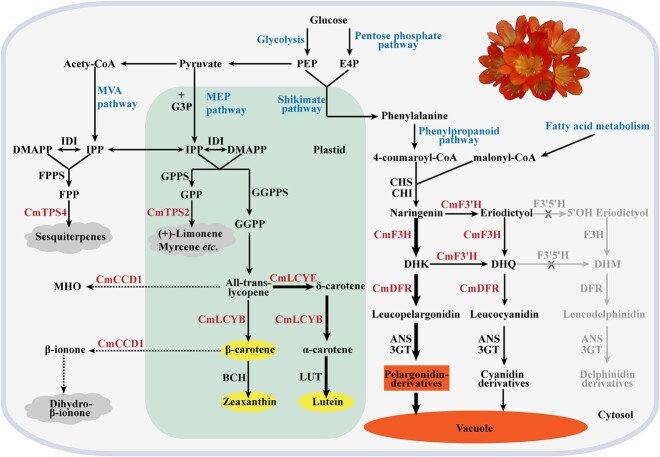
Simplified schematic diagram of the biosynthesis of the main specialized metabolites stored or secreted by *C. miniata* floral cells. Major pathway names are shown in blue and key enzymes characterized in the present study in red; stored or secreted anthocyanins, carotenoids, and terpenes are on a red, yellow and gray background, respectively. Metabolic routes are projected onto the subcellular localizations of the enzymes either characterized in the present study or referred to in other studies. Solid arrows indicate established biochemical reactions. Dotted arrows indicate hypothetical reactions. A single arrow does not necessarily represent a single enzymatic conversion. Arrow thickness indicates the relative content of metabolic flux at the bifurcation. MVA, mevalonate; IPP, isopentenyl pyrophosphate; DMAPP, dimethylallyl diphosphate; IDI, isopentenyl diphosphate isomerase; FPPS, farnesyl diphosphate synthase; FPP, farnesyl diphosphate; MEP, 2-c-methylerythritol 4-phosphate; G3P, glyceraldehyde 3-phosphate; GPPS, geranyl diphosphate synthase; GPP, geranyl diphosphate; GGPPS, geranylgeranyl diphosphate synthase; GGPP, geranylgeranyl diphosphate; TPS, terpene synthase; BCH, carotenoid β-hydroxylase; LUT, carotene ε-monooxygenase; MHO, 6-methyl-5-hepten-2-one; LCYB, lycopene β-cyclase; LCYE, lycopene ε-cyclase; CCD, carotenoid cleavage dioxygenase; PEP, phosphoenolpyruvate; E4P, erythrose 4-phosphate; CHS, chalcone synthase; CHI, chalcone isomerase; F3H, flavanone 3-hydroxylase; F3′H, flavonoid 3′-hydroxylase; F3′5′H, flavonoid 3′,5′-hydroxylase; DFR, dihydroflavonol 4-reductase; ANS, anthocyanidin synthase; 3GT, flavonoid 3-*O*-glucosyltransferase.

In conclusion, the molecular basis of floral color and scent of *C. miniata* was preliminarily investigated in the present study. To simplify, we have illustrated a model integrating anthocyanin, volatile terpene, and carotenoid pathways and proposed a scheme for further breeding *C. miniata* by molecular design (Fig. 7). Undoubtedly, the study provides foundations for thorough elucidation of the color and scent pathways in the *Clivia* genus, and clearly more work, such as studies integrating genome based multi omics [76, 77], is still needed. In addition, genes identified herein have the potential for usage in the improvement of new varieties of *C. miniata* as well as other ornamental plants.

## Materials and methods

Due to space constraints, the routine descriptions of some materials and methods are included in the supplementary Materials and methods, viz., Plant materials and growth conditions; Anthocyanin, terpene and carotenoid analysis; DNA and RNA extraction and cDNA synthesis; Primers used in the study (Supplementary Data Table S4) Gene and promoter cloning; Sequence alignment and phylogenetic analysis; and Quantitative real-time PCR analysis.

### Heterologous expression of *C. miniata* proteins in *E. coli*

Protein expression and extraction from prokaryotic cells were carried out following earlier studies [55]. Briefly, the ORFs of *CmF3H*, *CmDFR*, and *CmTPS2* were subcloned into BamHI- and EcoRI-digested pET32a vector with a Minerva Super Fusion Cloning Kit (US Everbright Inc., Suzhou, China). The certified vector was transformed into *E. coli* strain BL21 (DE3). The transformants were pre-cultured in 3 mL of LB medium at 37°C overnight. The pre-cultured transformants were inoculated into 300 mL of fresh medium and cultured at 37°C until an OD_600_ of 0.6. Subsequently, 0.2 mM isopropyl-β-d-thiogalactopyranoside (IPTG) was added to induce recombinant proteins at 16°C for 28 hours. Afterwards, the cells were harvested by centrifugation and resuspended in phosphate-buffered saline (PBS, pH 7.4) followed by sonication. The supernatant was then gathered by centrifugation at 13 225 g for 20 minutes at 4°C and further processed using an Ni-TED 6FF (Pre-Packed Gravity Column, Sangon Biotech, Shanghai, PRC). An imidazole gradient in PBS was employed to elute and purify the crude proteins. The imidazole in the purified proteins was removed by PBS dialysis at 4°C. The desalted proteins were then concentrated using a Silica Gel Dryer (Sangon Biotech, Shanghai, PRC) and their concentrations were quantified with a NanoDrop 1000 Spectrophotometer before further enzyme assays. For the heterologous expression of CmCCDs, previously used pACCAR25ΔcrtX, pACCAR16ΔcrtX, and pACCRT-EIB vectors were pre-transformed into *E. coli* strain BL21 (DE3) to generate engineered strains accumulating zeaxanthin, β-carotene, and lycopene, respectively [78]. Subsequently, the *CmCCD*s ligated in pET32a were introduced into the engineered *E. coli* strains. The subsequent pre-culture and inducing processes were the same as those mentioned above.

### Enzyme assay

The CmF3H1 activity assay was carried out following a published method with some modifications [79]. A total of 200 μL of reaction mixture contained 100 mM tricine (pH 7.5), 1 mM dithiothreitol, 1 mM FeSO_4_, 1 mg mL^−1^ ascorbic acid, 1 mM 2-oxoglutaric acid, 0.1 mg mL^−1^ bovine serum albumin, 5 μg of naringenin or eriodictyol (all from Solarbio, Beijing, PRC), and 50 μg of purified CmF3H1 protein. The enzyme assays of CmDFR1 and CmDFR2 were conducted as described earlier [55]. A 200-μL reaction mixture consisted of 100 mM Tris-HCl (pH 7.0), 2 mM NADPH, 5 μg of DHK, DHQ, or DHM (all from Solarbio, Beijing, PRC), and 50 μg of purified CmDFR1 or CmDFR2 protein. All the mixtures were incubated at 30°C for 30 minutes followed by two extractions in ethyl acetate. The upper solution was transferred to a new microtube and dried under vacuum. HPLC-grade methanol (30 μL) was added to resuspend the residues and HPLC analysis was performed using an ACCHROM XUnion C18 column. The column was eluted with different solvents. To analyze CmDFR proteins, we used isocratic elution with 40% solvent A (4% formic acid in water) and 60% solvent B (methanol). For CmF3H1, isocratic elution was carried out with 50% A (3% acetic acid in water) and 50% B (methanol). The flow rate was set at 1 mL minute^−1^ and detection was monitored at the UV absorbance wavelength of 290 nm.

Enzymatic assays of CmTPSs were performed following the method used in earlier studies [29]. In brief, 300 μL of mixture contained 50 mM HEPES buffer (pH 7.4), 7.5 mM MgCl_2_, 5 mM dithiothreitol, 5% glycerol, 60 μg protein, and 2 mM substrate (GPP and (*E*,*E*)-FPP from Sigma–Aldrich, NPP and (*Z*,*Z*)-FPP from Echelon Biosciences). The mixture was kept at 30°C for 2 hours. Volatile products were analyzed as shown in the section Anthocyanin, terpene and carotenoid analysis in the supplementary files. All the detection experiments were repeated at least three times for confirmation.

### Plant transformation

The ORFs of *C. miniata* anthocyanin biosynthetic genes were seamlessly cloned into BamHI and SacI digested pBI121 vector using the Minerva Super Fusion Cloning Kit and confirmed by sequencing. The constructed vectors were transformed into *Agrobacterium tumefaciens* strain GV3101 by the freeze–thaw method. The floral dip method was carried out to transform 5-week-old *Arabidopsis* [80]. The T2 transformants were screened by kanamycin and subjected to further analysis. Moreover, the *C. miniata TPS* genes were subcloned into NruI- and XhoI-processed pEAQ-HT vector [81]. The *Agrobacterium* strain harboring *CmTPS* or control vector was infiltrated into the leaves of 4-week-old *N*. *benthamiana*. Five days later, the infiltrated leaves were harvested and cut into pieces. Volatile terpenes from control and transgenic tobacco leaves were captured by silica fibers in an odor-free bottle and analyzed as described above. These experiments were repeated at least three times.

### Transient protoplast assay

The ORFs of *C. miniata* genes were seamlessly fused to vectors tagged with human influenza hemagglutinin (HA), GAL4 DNA binding domain (GD), green fluorescent protein (GFP), N-terminal 174 residues of GFP (GFPN) or C-terminal 66 residues of GFP (GFPC) by the primers in Supplementary Data Table S2 under the control of the cauliflower mosaic virus 35S promoter in the pUC19 backbone. The *C. miniata* promoters were cloned to promote the *GUS* (*beta-glucuronidase*) reporter gene in the modified pUC19 vector. Detailed information on the vectors and other constructs used in transient protoplasts can be found in our earlier studies [19, 46, 47]. All the newly constructed vectors were confirmed by being sequenced before being prepared with the GoldHi EndoFree Plasmid Maxi Kit (CWBIO, Beijing, PRC) according to the manufacturer’s instructions. Protoplast isolation, transfection, and reporter gene detection are described in detail elsewhere [19, 46, 47].

## Acknowledgements

Vectors and engineered *E. coli* strains for zeaxanthin, β-carotene and lycopene biosynthesis were kindly shared by Professor Shan Lu (Nanjing University, Nanjing, China). This work was supported by the National Natural Science Foundation of China (31900252, 31972445) and the Department of Science and Technology of Jilin Province (20210101005JC, 20190201299JC). The funders had no role in study design, data collection and analysis, decision to publish, or preparation of the manuscript.

## Author contributions

Y.L., R.G., J.Z., Y.W., P.K., K.L., A., M.L., and F.A. performed the experiments and helped in analyzing data. Y.L. drafted and revised the manuscript together with X.G. X.G. designed the experiments and discussed with L.W. and C.Z. All authors have participated in this research and approved the final manuscript.

## Data availability

RNA-seq data have been deposited in the NCBI repository (PRJNA813401). Other data supporting the findings of this study are available within the paper or its supplementary data.

## Competing financial interests

We declare that this research was conducted in the absence of any commercial or financial relationships that could be construed as a potential conflict of interest.

## Supplementary data

Supplementary data is available at *Horticulture Research* online.

## Supplementary Material

Web_Material_uhac114
